# Dendritic Cell Vaccination in Metastatic Melanoma Turns “Non-T Cell Inflamed” Into “T-Cell Inflamed” Tumors

**DOI:** 10.3389/fimmu.2019.02353

**Published:** 2019-10-09

**Authors:** Jenny Bulgarelli, Marcella Tazzari, Anna Maria Granato, Laura Ridolfi, Serena Maiocchi, Francesco de Rosa, Massimiliano Petrini, Elena Pancisi, Giorgia Gentili, Barbara Vergani, Filippo Piccinini, Antonella Carbonaro, Biagio Eugenio Leone, Giovanni Foschi, Valentina Ancarani, Massimo Framarini, Massimo Guidoboni

**Affiliations:** ^1^Immunotherapy–Cell Therapy and Biobank Unit, Istituto Scientifico Romagnolo per lo Studio e la Cura dei Tumori (IRST) IRCCS, Meldola, Italy; ^2^Unit of Biostatistics and Clinical Trials, Istituto Scientifico Romagnolo per lo Studio e la Cura dei Tumori (IRST) IRCCS, Meldola, Italy; ^3^Dipartimento di Medicina e Chirurgia, Università degli Studi di Milano-Bicocca, Milan, Italy; ^4^Scientific Directorate, Istituto Scientifico Romagnolo per lo Studio e la Cura dei Tumori (IRST) IRCCS, Meldola, Italy; ^5^Department of Computer Science and Engineering (DISI), University of Bologna, Bologna, Italy; ^6^Advanced Oncological Surgery Unit, Morgagni-Pierantoni Hospital, Forlì, Italy

**Keywords:** melanoma, tumor microenvironment, T cell landscape, dendritic cell vaccine, immunomonitoring, immunotherapy, PDL1

## Abstract

Dendritic cell (DC)-based vaccination effectively induces anti-tumor immunity, although in the majority of cases this does not translate into a durable clinical response. However, DC vaccination is characterized by a robust safety profile, making this treatment a potential candidate for effective combination cancer immunotherapy. To explore this possibility, understanding changes occurring in the tumor microenvironment (TME) upon DC vaccination is required. In this line, quantitative and qualitative changes in tumor-infiltrating T lymphocytes (TILs) induced by vaccination with autologous tumor lysate/homogenate loaded DCs were investigated in a series of 16 patients with metastatic melanoma. Immunohistochemistry for CD4, CD8, Foxp3, Granzyme B (GZMB), PDL1, and HLA class I was performed in tumor biopsies collected before and after DC vaccination. The density of each marker was quantified by automated digital pathology analysis on whole slide images. Co-expression of markers defining functional phenotypes, i.e., Foxp3^+^ regulatory CD4^+^ T cells (Treg) and GZMB^+^ cytotoxic CD8^+^ T cells, was assessed with sequential immunohistochemistry. A significant increase of CD8^+^ TILs was found in post-vaccine biopsies of patients who were not previously treated with immune-modulating cytokines or Ipilimumab. Interestingly, along with a maintained tumoral HLA class I expression, after DC vaccination we observed a significant increase of PDL1^+^ tumor cells, which significantly correlated with intratumoral CD8^+^ T cell density. This observation might explain the lack of a significant concurrent cytotoxic reactivation of CD8^+^ T cell, as measured by the numbers of GZMB^+^ T cells. Altogether these findings indicate that DC vaccination exerts an important role in sustaining or *de novo* inducing a T cell inflamed TME. However, the strength of the intratumoral T cell activation detected in post-DC therapy lesions is lessened by an occurring phenomenon of adaptive immune resistance, yet the concomitant PDL1 up-regulation. Overall, this study sheds light on DC immunotherapy-induced TME changes, lending the rationale for the design of smarter immune-combination therapies.

## Introduction

According to the cancer-immunity cycle, dendritic cells (DCs) play a fundamental role in setting off an anti-tumor specific immune response (1). Indeed, under ideal circumstances, DCs take up tumor antigens and promote the generation of anti-tumor specific T cells, which ultimately infiltrate the tumor bed and kill their target cells through cytolytic mechanisms (i.e., perforin and granzyme B). The translation of this concept into the clinic has led to the design of DC-based therapeutic vaccines (2). Since their first utilization, many clinical trials have been conducted in metastatic melanoma patients accounting for an objective response rate of 8.5%, as reported in a meta-analysis conducted in 1,205 advanced melanoma patients treated with DC vaccination monotherapy (3). Similar data were observed in our long-term follow-up series (4). Delayed-type hypersensitivity skin test (DTH) and quantification of peripheral antigen-specific anti-tumor T cell response with Enzyme-Linked immunoSPOT assay and/or tetramer analysis on peripheral blood samples, longitudinally collected during the treatment, are commonly used to evaluate the immunogenicity of DC vaccines. However, the reported induced tumor-specific immune responses measured in the blood seem to only partially correlate with efficacy (5). A higher enrichment of antigen specific T cells inside the tumor compared to the blood has been argued as a potential consequence of this phenomenon (6, 7). Intriguingly, it was also suggested that the presence of an anti-tumor specific blood response, even if weak, could serve to promote a local inflammation in the tumor microenvironment (TME), and intratumoral CD8^+^ CTL infiltration has also been envisaged as the clinically relevant consequence of the eosinophilia found in DC vaccinated patients and associated with positive outcome (8). However, notwithstanding the recognized importance of the TME, no studies have been conducted to assess whether an *in situ* analysis could allow adding additional insight into the local immune modulation occurring in DC vaccinated patients. Besides systemic anti-tumor immunity, it becomes now clear that the immune contexture holds precious information endowed with clinical impact (9). In particular, the content of intratumoral immune cells, especially CD8^+^ T cells, strictly correlates with patients' prognosis (10) across different tumor types, melanoma included (11). Accordingly, immunological characterization of the TME along treatment is increasingly utilized for identifying biomarkers of response and mechanisms of resistance to cancer immunotherapies (12, 13). Evidence available from the literature has been primarily obtained in patients treated with immune checkpoint inhibitors (14, 15), and aimed at identifying biomarkers predictive of response to therapy and finding potentially actionable synergistic targets for improving their clinical efficacy. In this line, clinical experience with combined anti-CTLA4 and anti-PD1 immunomodulating antibodies has shown better efficacy in melanoma patients, but at the expenses of more severe treatment-related toxicities. DC immunotherapy has a robust safety profile, which makes it an interesting good candidate for better-tolerated combination immunotherapies. Nonetheless, an extensive characterization of changes occurring in the TME upon DC vaccination is currently lacking. In order to fill this gap in the literature, we addressed by immunohistochemical (IHC) analysis the local modulation of the T cell landscape upon DC vaccination in a retrospective series of matched pre and post formalin-fixed paraffin embedded (FFPE) tumor lesions collected from a series of 16 metastatic melanoma patients treated with autologous DCs loaded with tumor lysate/homogenate. Our data show a DC vaccine-induced modulation of the TME, with the emergence of changes suggestive of a T cell inflamed TME, i.e., a robust CD8^+^ T cell infiltration along with the up-regulation of PDL1. Altogether, our findings support the use of DC immunotherapy as a TME modulating therapeutic tool, which might broaden the effectiveness of anti-PD1/PDL1 therapies.

## Materials and Methods

### Patients

In this study, we evaluated 16 patients with metastatic melanoma enrolled in different vaccination protocols from 2000 to 2015. All patients were given intradermally mature autologous dendritic cell pulsed with autologous tumor lysate (ATL) or autologous tumor homogenate (ATH) and keyhole limpet hemocyanin (KLH). DC vaccine was administered alone (mainly in a compassionate use program, CUP) or combined with different conditioning therapies, e.g., low doses of temozolomide prior to the vaccine (16) or INF alpha before leukapheresis (17) as described in Table 1. Pre-treatment tumor samples were obtained from tumor lesions surgically removed for the preparation of ATL or ATH. Post-vaccine biopsies were obtained for diagnostic and/or therapeutic purposes and were taken at least after the fourth induction dose of the vaccine. The median time from the pre-treatment biopsies to therapy was 3 months (0–29 months, average 6.25 months). All post-therapy lesions have been collected on-treatment besides Pt#2, Pt#6, and Pt#10 for whom the tumor was sampled after 18, 7, and 3 months from the last vaccine dose, respectively. The median time from start to biopsy was 5 months for the on-treatment samples (3–24 months, average 7 months) and 7 months for the post-treatment samples (3–18 months, average 9.33 months). Clinical response was defined according to RECIST 1.1 criteria (18) and surgically removed post-vaccine tumor lesions were classified as regressing if changes in their longest diameter were ≥–30% compared with the baseline, stable if changes were comprised between −30 and +20%, and progressing if ≥+20% (Table 1). All patients gave their informed consent to the study, which was conducted in accordance with the Declaration of Helsinki following a protocol approved by the local Institutional Review Board.

**Table 1:** Patients' characteristics (*n* = 16).

**Pt#ID**	**BRAF status**	**Vax protocol**	**Tumor lesion, site**		**Months from surgery to first vax**	**Months from baseline vax to biopsy harvesting (on-treatment or post-treatment biopsy)**	**BOR (RECIST)/ duration**	**OS**	**Objective response of post-vax biopsy**	**DTH**	**Previous/ following treatments**	**Ratio post/ pre CD8 (cells/mm^**2**^)**	**PDL1 in total cells (%)**	
			**Baseline**	**Post-vax**									**Pre**	**Post**
***1#***	**V600E**	**CUP**	**Lymph node**	**Lymph node**	**1**	**17 (on-treatment)**	**CR/8**	**34**	**Progressing**	**+**	**No/CT, High dose IL-2**	**1,101**	**7,836**	**3,022**
*2#*	V600E	CUP	Lymph node	Subcutis	15	18 (post-treatment)	PR/68	87	Stable	+	BioCT/Surgery	0,913	0,726	0,402
*3#*	V600E	CUP	Omentum	Stomach	2	24 (on-treatment)	SD/50	108+	Stable	+	BioCT/Surgery,RT	0,823	16,750	6,185
*4#*	WT	Tem	Peritoneum	Subcutis	2	4 (on-treatment)	SD/4	16	Progressing	+	CT, Ipi/RT	0,173	4,744	4,793
***5#***	**na**	**Tem**	**Subcutis**	**Omentum**	**1**	**4 (on-treatment)**	**SD/10**	**45**	**Progressing**	**+**	**RT/Ipi**	**1,338**	**11,130**	**24,700**
*6#*	V600E	Tem	Lung	Skin	3	7 (on-treatment)	SD/9	62	Progressing	+	BioCT/low doses IL-2, Ipi	0,521	0.9748	9,203
***7#***	**V600E**	**CUP**	**Subcutis**	**Subcutis**	**10**	**5 (post-treatment)**	**SD/7**	**22**	**Progressing**	**+**	CT*/Ipi	**0,560**	**0,826**	**7,519**
***8#***	**WT**	**CUP**	**Jejunum**	**Adrenal gland**	**29**	**4 (on-treatment)**	**PR/57**	**87+**	**Stable**	**++**	**CT/Ipi, CT**	**3,010**	**0,000**	**33,639**
***9#***	**V600E**	**CUP**	**Lymph node**	**Subcutis**	**3**	**5 (on-treatment)**	**SD/6**	**23**	**Progressing**	**+**	**No/ Ipi,vemurafenib, Tem**	**4,039**	**0,602**	**6,530**
*10#*	V600E	CUP	Subcutis	Lymph node	12	3 (post-treatment)	SD/5	18	Progressing	+	Biot/RT	0,110	8,771	na
***11#***	**WT**	**CUP**	**Subcutis**	**Subcutis**	**3**	**9 (on-treatment)**	**SD/5**	**27**	**Progressing**	**+**	**No/CT, Ipi**	**1,051**	**1,255**	**13,999**
*12#*	WT	CUP	Subcutis	Subcutis	8	4 (on-treatment)	PD^¥^	13	Progressing	-	Biot/No	1,355	na	na
***13#***	**WT**	**Vax+INFα**	**Subcutis**	**Subcutis**	**5**	**3 (on-treatment)**	**PD**	**11**	**Progressing**	**+**	**No/Ipi**	**2,260**	**1,677**	**8,201**
***14#***	**WT**	**Vax+INFα**	**Lymph node**	**Subcutis**	**0**	**3 (on-treatment)**	**PD**	**7**	**Progressing**	**+**	**No/Ipi**	**5,013**	**2,465**	**11,765**
*15#*	WT	Vax only	Subcutis	Brain	4	6 (on-treatment)	SD/5	8	Progressing	+	CT, Ipi/No	1,080	2,379	0,893
***16#***	**V600E**	**CUP**	**Adrenal gland**	**Skin**	**2**	**5 (on-treatment)**	**PD**	**19**	**Progressing**	**+**	**No/Ipi, vemurafenib, BioCT, pembro**	**5,097**	**1,709**	**14,279**

Vax, dendritic cell vaccination; DTH, delayed-type hypersensitivity; WT, wild type; CUP, compassionate use program; CT, chemotherapy; CT^*^, chemotherapy third line; RT, radiotherapy; Tem, temozolomide; IFNα, interferon alpha; IL-2, interleukine 2; Biot, low doses of cytokines (IL-2 and/or IFNα); BioCT, cytokine+chemotherapy; Ipi, ipilimumab; pembro, pembrolizumab. Naïve/CT/RT patients are highlighted in bold. ^¥^Pt#12 was treated beyond progression after surgical removal of the progressing lesion.

### Generation and Administration of DCs

DCs were prepared following Good Manufacturing Practice (GMP) guidelines and according to Ridolfi et al. (16). Briefly, monocytes obtained by adherence of the leukapheresis product on culture flasks were cultured in CellGro DC medium supplemented with interleukin 4 (IL-4, Cell Genix) and granulocyte-macrophage colony stimulating factor (GM-CSF, Cell Genix) for 7 days. On day 6, 90% of the DC culture was pulsed with ATL or ATH (100 mg/ml), while the remaining 10% was pulsed with KLH (50 mg/ml). On day 7, the culture medium was discarded and immature DCs were cultured for further 2 days with a maturation cocktail comprising the following cytokines: TNFα, IL-1β, IL-6 (Cell Genix), and PGE2 (Pfizer). On day 9, mature dendritic cells (median 10^7^, range 2.2–20.8 × 10^6^) were recovered, washed, suspended in sterile saline solution and immediately injected. As part of our standard release criteria, before administration, DCs were checked for safety (sterility, mycoplasma, endotoxin), vitality, purity, and maturation phenotype. Purity was always reported to be >60% (average 63, 59%). The maturation phenotype of the infused DCs was confirmed by flow cytometry using the following markers: HLADR (accepted cut-off value ≥60%, average = 85, 8%), CD80 (accepted cut-off value ≥50%, average = 83, 4%), CD83 (accepted cut-off value ≥40%, average = 74,9%), and CD86 (accepted cut-off value ≥60%, average = 78,8%). Patients were given 10^7^ DCs intradermally at the base of the thigh or groin every 2 weeks for 4 cycles, followed by monthly doses until progression, worsening of clinical conditions (ECOG performance status > 2), or autologous tumor lysate shortage. Patients who ran out of tumor lysate, but had additional surgically removable tumor lesions, were retreated utilizing tumor lysate obtained from newly removed lesions. Antitumor immune response to the DC vaccine was evaluated with Delayed-Type Hypersensitivity (DTH) test as follows: serial concentrations (100, 50, 20, 10, and 5 μg) of autologous tumor lysate and KLH were intradermally injected into the forearms of patients and erythema and induration were recorded after at least 24 h. DTH was considered as positive if the area of erythema and/or induration measured at least 5 mm at any antigen concentration.

### Standard and Sequential Immunohistochemistry (IHC)

IHC stainings were performed on 4 μm FFPE tissue sections. Briefly, after deparaffinization in xylene and rehydration in graded ethanol, sections were washed in phosphate-buffered saline (PBS). After an antigen retrieval step, sections were incubated with the primary antibody (see Table 2 for details). Reactions carried out on Ventana BenchMark automated slide stainer were performed according to manufacturer's instructions. The sections were then kept for 15 min at room temperature (RT) before further PBS washing and immunostained with a standard streptavidin-biotin-peroxidase procedure, followed by a 3,3-diaminobenzidine (DAB) color reaction and counterstaining with hematoxylin. Experimental conditions and list of primary antibodies clones are reported in Table 2. All the antibody conditions were validated on tissue microarrays (TMAs) containing different positive control tissues. For sequential IHC, a non-biotin Poly HRP conjugate system followed by aminoethyl carbazole (AEC) substrate reaction was used instead of DAB. For consecutive cycles of staining, a chromogen destaining step (in alcohol) and a stripping step (in citrate buffer) were applied according to a previously published protocol (19). Reproducibility of the staining along increasing cycles of staining/destaining was checked for each marker utilized in sequential staining (Supplementary Figures 1, 2).

**Table 2:** Details of IHC antibodies.

**Antibody**	**Clone**	**Isotype /host**	**Supplier cat#**	**Dilution/Ab reaction**	**Antigen retrieval**	**Ab diluent**	**Position in sequential IHC/AEC reaction time**
CD45	2B11+ PD7/26	IgG1/Mouse monoclonal	Dako Cat#M0701	1:50/1 h RT	EDTA (Ph8) water bath 100°C, 40 min	PBS + 1%BSA + 0,02% sodium azide	2°/30 min
CD4	**EPR6855**	**IgG/rabbit monoclonal**	**Abcam Cat#ab133616**	**1:100/1 h RT**	**EDTA (Ph8) water bath 100°C, 40 min**	**PBS** **+** **1%BSA** **+** **0,02% sodium azide**	**4°/30 min**
	4B12	IgG1/mouse monoclonal	Dako Cat#M7310	1:100/1 h RT	TRIS EDTA (Ph9) water bath 98.5°C, 20 min	Ventana Antibody Diluent Cat#251-018	
CD8	**4B11**	**IgG2b/mouse monoclonal**	**Thermo scientific Cat#MA1-80231**	**1:40/1 h RT**	**EDTA (Ph8) water bath 100°C, 40 min**	**PBS+1%BSA+** **0,02% sodium azide**	**4°/30 min**
	4B11	IgG2b/mouse monoclonal	Novocastra Cat#NCL-L-CD84B11	1:100/1 h RT	TRIS EDTA (Ph9) water bath 98.5°C, 20 min	Ventana Antibody Diluent Cat#251-018	
Foxp3	**SP97**	**IgG/rabbit monoclonal**	**Thermo Scientific Cat#MA5-16365**	**1:100/1 h RT**	**EDTA (Ph8) water bath 100°C, 40 min**	**PBS** **+** **1%BSA** **+** **0,02% sodium azide**	**2°/30 min**
	236A/E7	IgG1/mouse monoclonal	Abcam Cat#ab20034	1:100/1 h RT	Citrate buffer (Ph6) water bath 98.5°C, 20 min	Ventana antibody diluent Cat#251-018	
Granzyme B	GrB-7	IgG2a/mouse monoclonal	Merk/Millipore Cat#MAB3070	**1:20/1 h RT**	**EDTA (Ph8) water bath 100°C, 40 min**	**Ventana Antibody Diluent Cat#251-018**	**1°/30 min**
				1:20/1 h RT	Citrate buffer (Ph6) water bath 98.5°C, 20 min	Ventana Antibody Diluent Cat#251-018	
PDL1	SP142	IgG/rabbit monoclonal	Spring Bioscience Cat#M4424	**1:100/1 h RT**	**EDTA (Ph8) water bath 100°C, 40 min**	**PBS** **+** **1%BSA** **+** **0,02% sodium azide**	**1°/30 min**
				1:40/1 h RT	Cell conditioning solution (CC1) ventana BenchMark Cat#950-124	Ventana antibody diluent Cat#251-018	

*Antibodies and conditions applied in the optimized sequential IHC protocol are indicated in bold*.

### Image Acquisition

High-resolution whole slide images (40x and 20x magnifications) (WSI) of IHC stained slides were acquired using the Aperio CS2 slide scanner (Leica Biosystems Nussloch GmbH) or the MicroVisioneer Manual WSI system (MicroVisioneer, 20x magnification).

### Software Assisted Quantification of Single IHC Stains

Digital pathology analysis was performed on WSIs with *QuPath*, an open source image analysis software (20). Quantification of IHC stains was supervised by an expert Pathologist (MG): tumor areas, non-tumoral stroma, and necrotic areas were separately annotated, and artifacts (e.g., tissue folding) deselected and excluded from the analysis. Quantification of marker positive cells was performed in tumor areas, excluding non-tumoral stroma and necrosis. In the analysis of the lymph node metastasis (*n* = 5) attention was paid to only count for tumor-infiltrating T lymphocytes, excluding those associated to the lymph node tissues. The density of positive cells (i.e., the number of positive cells per mm^2^) was calculated for CD4, CD8, FoxP3 and Granzyme B; the percentage of positive cells on the total was instead calculated for PDL1. Detection of positive cells was performed using *QuPath's Simple Tissue Detection* and *Positive Cell Detection* methods. Briefly, bright-field images were analyzed using the setup parameter *optical density sum* to avoid nuclei detection loss in samples showing weak haematoxylin counterstain. Alternatively, the *Hematoxylin OD* setup parameter was preferentially used to avoid overestimating the total cell number (due to background artifacts). For nuclear markers detection (e.g., Foxp3) the AEC or DAB signal was assessed using the command *Nucleus DAB OD mean* or *max*, whereas *Cell DAB OD mean* or *Cytoplasm DAB OD mean* commands were applied for surface or cytoplasmatic markers detection, respectively. Otherwise, when active the *Optical density sum* parameter, the intensity of the cell and cytoplasmatic signal was assessed in the cell nuclei, since this parameter tends to include in the nucleus the AEC or DAB signal that comes from the membrane or cytoplasm. Parameters were set-up for each slide on at least three fields selected for optimal or suboptimal staining (e.g., high melanin content) to obtain the better parameters combination for total cells and positive cells enumeration. Then, the number of positive cells detected per area was used to calculate the average number of positive cells per mm^2^, and these results exported along with mark-up images showing the detected cells for visual verification.

### Processing of Multiplex/Sequential IHC Images

To perform co-localization analyses, we designed *Data Science for Health (DS4H) Image Alignment*, a user-friendly tool freely provided as an *ImageJ/Fiji* plugin. With *DS4H Image Alignment*, multiplex/sequential IHC images can be easily co-registered by defining with a few clicks some well-visible reference marks. The implemented least-squares method automatically approximates the solution of the mathematical over-determined system, so to define the registration matrix then used to align the different images (21, 22). It also considers rotations and scale changes in case the staining/destaining/stripping steps generated a tissue dilation/shrink (23). Finally, it provides an iterative subroutine for a fine alignment, to easily reach a very good image co-registration quality. Practically speaking, the sequential IHC images considered in this work have been: (a) imported into *Fiji*; (b) cropped to extract corresponding, significant Regions Of Interest (ROI); (c) aligned with *DS4H Image Alignment*; (d) separated into single channels using the *H AEC* option of the *ImageJ/Fiji Color Deconvolution* tool (24); then, (e) the AEC channels have been re-aligned into a *z*-stack for final comparisons. *DS4H Image Alignment* has been implemented in *Java* as a plugin for *ImageJ/Fiji*. It works with “*.svs*” files, but also all the medical imaging formats included in *Bio-Formats* (25, 26). *DS4H Image Alignment* version 1.0 is freely available at: www.filippopiccinini.it/DS4H-IA.html, together with a sample dataset and a video tutorial.

### Statistical Analysis

Statistical analysis was performed using GraphPad Prism (version 6, Jolla, CA, USA). A non-parametric two-tailed Wilcoxon signed-rank test was used to evaluate differences in the distribution of the number of cells positive for a given marker per square millimeter between pre- and post-vaccine biopsies. Correlations of PDL1 expression with the immune infiltrate were analyzed by Spearman's rank correlation coefficient. A *p*-value of <0.05 was considered as statistically significant.

## Results

### Patients and Clinical Outcomes

Based on the availability of both pre- and post-vaccine tumor biopsies, 21 patients treated with DC vaccination at Morgagni Hospital (Forlì, FC, Italy) and IRCCS-IRST (Meldola, FC, Italy) between 2000 and December 2015 were initially selected: in five cases pre-treatment or post-treatment biopsies were not evaluable (mainly for insufficient tumor tissue left). Detailed information on patient characteristics and vaccine administration is provided in Table 1. All patients, except one (Pt#12), were immunoresponsive to the vaccine, as shown by positivity to DTH tests performed after at least four induction immunizations. Patients' median age at study entry was 51 years (range 31–73) and both genders were equally represented (nine males and seven females). In 11 out of the 16 cases indagated the sites of tumor biopsies taken before and after vaccination were of the same type, i.e., soft tissue/nodal or visceral, according to the classification provided in Bartlett et al. (27), thus avoiding any statistically significant imbalance in the level of CD8 expression in the selected pre-treatment sample cohorts. Best overall response (BOR) to the treatment per RECIST 1.1 criteria was complete response (CR) in one patient, partial response (PR) in two patients, stable disease (SD) in nine patients, while the remaining four patients showed progressive disease (PD) at the first radiological tumor assessment. Retrospective evaluation confirmed that Pt#12, Pt#13, Pt#14, and Pt#16 were all confirmed PD even when immune-related response criteria were applied. Median duration of response was 7.5 months (range 4–68 months). Median overall survival was 22 months (range 7–108 months). Six patients were given DC vaccine as a first line therapy, whereas the remaining had received at least one therapy line before (chemotherapy, radiotherapy, biochemotherapy or immunotherapy).

### Increase of CD8^+^ T Cell Characterizes Post-DC Vaccine Tumor Lesions of Naïve and Chemo/Radiotherapy Treated Melanoma Patients

**Figure 1:**
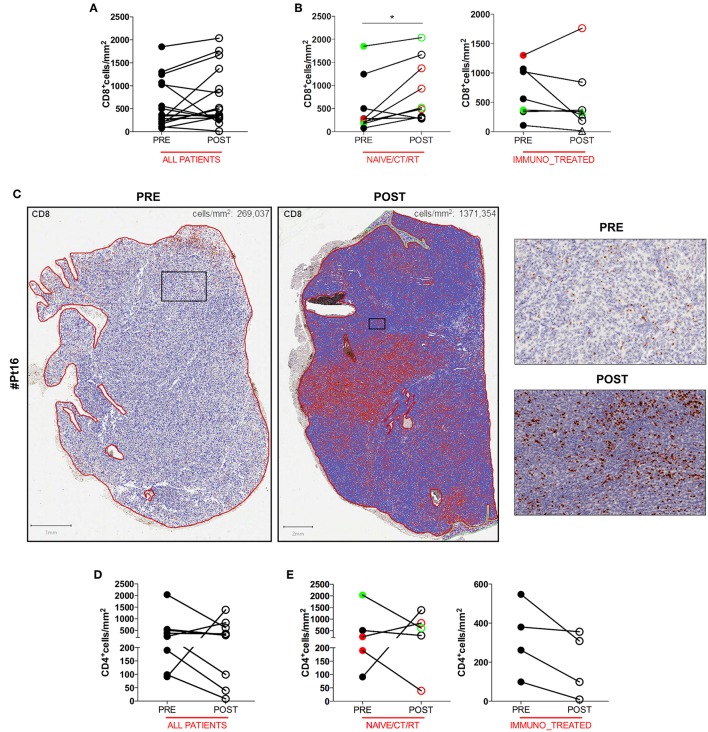
Quantitative analysis of intratumoral CD8^+^ and CD4^+^ T cells of serial tumor biopsies from DC vaccine treated patients. The number of intratumoral CD8^+^ T cells per mm^2^ in matched pre- and post- treatment samples is plotted in graphs. Graph showing *all patients* (*n* = 16, *p* = 0.2144) **(A)**. Right and left graphs showing *naïve/CT/RT* (*n* = 9, *p = 0.0195), and *immuno_treated* (*n* = 7, *p* = 0.2969), respectively **(B)**. Whole slide images (WSI) of CD8 staining in the pre- and post-vaccine lesions of one representative patient (Pt#16). Scale bars 1 and 2 mm for the left and the right WSI panel, respectively. Higher magnification images for the mark-up CD8 stain in pre- and post- treatment samples are shown. Scale bars, 100 μm **(C)**. The number of intratumoral CD4^+^ T cells per mm^2^ in matched pre- and post- treatment samples is plotted in graphs. Graph showing *all patients* (*n* = 9, *p* = 0.4258) **(D)**. Right and Left graphs showing *naïve/CT/RT* (*n* = 5, *p* > 0.9999) and *immune_treated* (*n* = 4, *p* = 0.1250), respectively **(E)**. CR and PR Patients are displayed in green, SD in black, and PD in red. Open circles denote on-treatment samples, open triangles post-treatment ones. Statistical comparisons are based on the non-parametric two-tailed Wilcoxon signed-rank test. Only values statistically significant are reported: *p < 0.05.

To gain insight into the intratumoral T cell landscape of DC vaccinated patients, quantification of CD8 and CD4 positive cells was performed on matched pre and post FFPE tumor biopsies. Globally, the amount of intratumoral CD8^+^ T cells increased in post-treatment tumor biopsies compared with pre-treatment ones, although this change did not reach statistical significance (mean ± SEM 597.9 ± 132.7 vs. 731.5 ± 159.0 CD8^+^ cells/mm^2^ in pre-treatment and post-treatment biopsies, respectively; *p* = 0.2114; Figure 1A). In six cases the content of intratumoral CD8^+^ T cells decreased after treatment: interestingly, one of these patients (Pt#4) started DC vaccination after failure on Ipilimumab and showed a very high pre-treatment level of intratumoral CD8^+^ T cells (1,068.927 cells/mm^2^ vs. 185.006 cells/mm^2^, in pre- vs. post-vaccine lesion, respectively; Table 1, Supplementary Table 1). Moreover, four out of the five remaining patients had previously received cytokines either in combination with chemotherapy (BiotCT) or as low doses IFNalpha and IL-2 (Biot), and showed higher levels of pre-treatment levels CD8^+^ T cells as well (Table 1, Supplementary Table 1). Along this line, patients were stratified into two separate groups: 1) *naïve/CT/RT*, comprising patients who received the vaccine as a first line therapy (*n* = 6), after chemo- (*n* = 2), or radiotherapy (*n* = 1); 2) *immuno_treated*, accounting for all patients (*n* = 7) previously treated with immunomodulating cytokines or anti-CTLA4 (Ipilimumab, Ipi). Intriguingly, a selective significant increase of tumor-associated CD8^+^ T cells in post- vs. pre-vaccine samples was observed in the *naïve/CT/RT group* (mean ± SEM 533.2 ± 201.7 vs. 878.6 ± 220.3 CD8^+^ cells/mm^2^ in pre-treatment and post-treatment biopsies, respectively; *p* = 0.0195, Figure 1B, Supplementary Table 1) compared to the *immuno_treated* one (mean ± SEM 681.0 ± 1,649.7 vs. 542.4 ± 224.9 CD8^+^ cells/mm^2^ in pre-treatment and post-treatment biopsies, respectively; *p* = 0.2969; Figure 1B, Supplementary Table 1). Figure 1C shows the *QuPath*-generated mark-up WSI of CD8 stain in the pre and post-vaccine lesions of Pt#16 displaying a remarkable increase in tumor-associated CD8^+^ T cells (269,037 vs. 1,371,354 cells/mm^2^, in pre- and post-vaccine, respectively). Of note, while lesions analyzed upon DC vaccination were mainly resected during treatment (on-treatment samples), as for all those of the *naïve/CT/RT group*, three out of seven samples belonging to the *immuno_treated group* (identified with square within the graphs) represent lesions sampled after the last vaccine dose (post-treatment samples). To reinforce our results and data interpretation we confirmed the absence of statistically significant difference in the post/pre CD8 ratio between these post-treatment samples (*n* = 3) and those harvested on-treatment (*n* = 4) (Mean ± SEM; 0.5147 ± 0.2318 and 0.8578 ± 0.2528, respectively).

No significant change in the number of intratumoral CD4^+^ T cells upon DC vaccination was observed in any of the patient groups (Figures 1D,E), speaking in favor of a specific DC vaccine-mediated modulation of the CD8^+^ Cytotoxic T Cell arm of the adaptive immune system.

### CD8^+^ T Cell Infiltration Is Paralleled by a Concurrent Increase of PDL1 Expression in Tumor Cells

**Figure 2:**
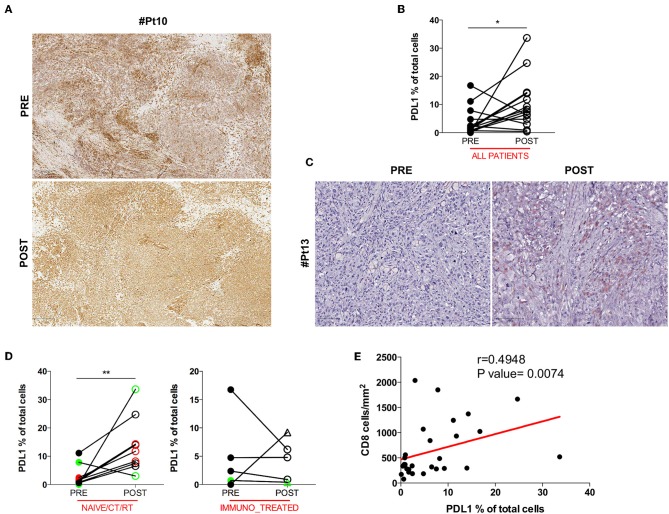
HLA class I and PDL1 expression in serial tumor biopsies from DC vaccine treated patients. The pattern of HLA class I expression by melanoma cells is shown in matched pre- and post-therapy lesions for one representative patient (Pt#10). Scale bars, 250 μm **(A)**. Differences in intratumoral PDL1 expression are illustrated in the graph as the percentage of PDL1 expressing cells on the total cell number (*n* = 14, *p = 0.0353) **(B)**. A representative example of the staining is shown (Pt#13). Scale bars, 100 μm **(C)**. PDL1 expression distribution in matched samples within the *naïve/CT/RT* cohort and the *immuno_treated* cohort is reported in right and left graphs (*n* = 9, **p = 0.0078 and *n* = 5, *p* = 0.6250), respectively. CR and PR Patients are displayed in green, SD in black, and PD in red. Open circles denote on-treatment samples, open triangles post-treatment ones **(D)**. A positive correlation was found between CD8 and PDL1 expression (Spearman *r* = 0.4948, *p* = 0.0074) **(E)**. Statistical comparisons are based on the non-parametric two-tailed Wilcoxon signed-rank test. Only values statistically significant are reported: *p < 0.05 and **p < 0.01.

Anti-tumor T cell activity requires antigenic presentation in the context of human leukocyte antigen (HLA) molecule and loss or inability to up-regulate HLA class I expression is a common mechanism of tumor immune escape. Another crucial way tumor cells avoid immune-mediated killing is the up-regulation of the immune checkpoint molecule PDL1. Therefore, we assessed whether the increase in intratumoral CD8^+^ T cells after DC vaccination was associated with relevant changes in the expression of HLA class I by melanoma cells or in the pattern of expression of PDL1. No difference was found in HLA class I expression between pre and post-DC therapy lesions (Figure 2A), indicating that loss/downregulation of HLA class I molecules is unlikely involved in immune escape after DC vaccine in our series. Intriguingly, when PDL1 expression was evaluated, a significant increase in the number of PDL1^+^ tumor cells was detected in post-vaccination tumor biopsies (11 out of 14 assessable paired biopsies, mean ± SEM 3.721 ± 1.316 vs. 10.37 ± 2.456 PDL1% in pre-treatment and post-treatment biopsies, respectively; *p* = 0.0353) (Figure 2B). A representative case is shown in Figure 2C. Of note, we found that this up-regulation was stronger in the *naïve/CT/RT cohort* (*n* = 9, mean ± SEM 3.056 ± 1.268 vs. 13.74 ± 3.242 PDL1% in pre-treatment and post-treatment biopsies, respectively; *p* = 0.0078), while it did not reach statistically significance in the *immune_treated one* (*n* = 5, mean ± SEM 4,920 ± 3,068 vs. 4,295 ± 1,653 PDL1% in pre-treatment and post-treatment biopsies, respectively; *p* = 0.6250), suggesting a positive correlation with the observed higher density of intratumoral CD8^+^ T cell infiltrate (Figure 2D). Indeed, Spearman's correlation analysis showed a significant positive correlation between the density of CD8^+^ T cells and the percentage of PDL1^+^ cells (Spearman *r* = 0.4948, *p* = 0.0074; Figure 2E). Mark-up WSI overview of PDL1 stain for Pt#16 (Supplementary Figure 3A) shows the spatial distribution of PDL1 expression in post-therapy biopsy and clearly highlights that the enriched expression of PDL1 was topographically associated with the CD8^+^ T cell infiltrated areas (Figure 1B). Sequential staining on the same tissue section of CD45 and PDL1 allowed discerning its relative expression on tumor cells and immune cells. Pseudo-fluorescence double images (Supplementary Figure 3B) confirmed that PDL1 was expressed almost exclusively in tumor cells and underscore the proximity of CD45^+^ immune cells to that of PDL1^+^ tumoral cells, stressing the inducible nature of PDL1 expression.

### Intratumoral PDL1 Counteracts Cytotoxic Activation of Intratumoral CD8^+^ T Cells

**Figure 3:**
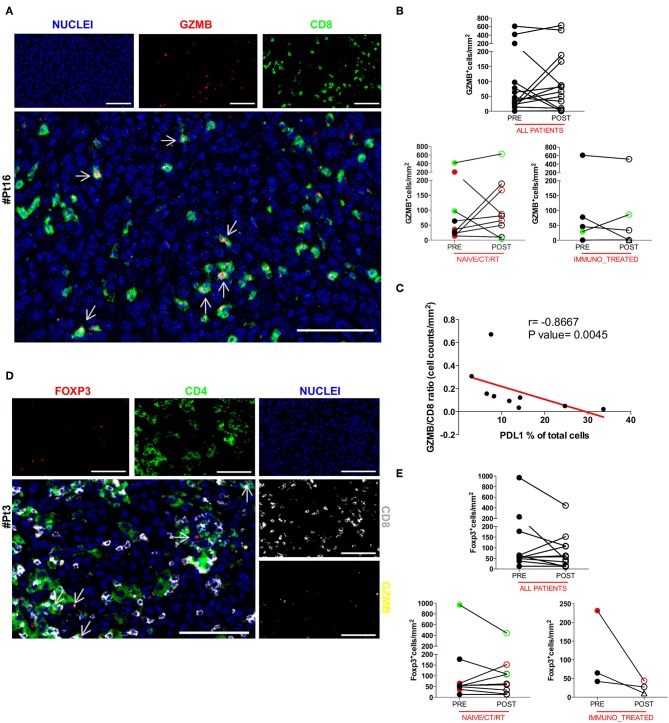
Analysis of the functional phenotype of intratumoral CD8^+^ and CD4^+^ T cells. The co-expression of GZMB and CD8 was evaluated by sequential IHC. AEC color signals were extracted from each digitized single-marker image by color deconvolution, followed by pseudo-coloring. A representative image is shown (Pt#16). Nuclei (blue), GZMB (red), CD8 (green). Scale bars, 100 μm **(A)**. The GZMB expression between matched pre- and post-therapy samples, either in the total patient cohort or in any of the defined patient groups is illustrated in graphs (*all patients n* = 14 *p* = 0.5830, *naïve/CT/RT n* = 9 *p* = 0.2500, *immuno_treated n* = 5 *p* = 0.4375) **(B)**. Correlation between the GZMB ÷ CD8 ratio and the percentage of PDL1 over total cells in the *naïve/CT/RT* patient cohort (Spearman *r* = −0.8667, **p = 0.0045) **(C)**. FFPE pre-therapy sections were analyzed by multiplex IHC. Results from a representative patient (Pt#3) are shown. Nuclei (blue), GZMB (yellow), CD8 (gray), CD4 (green), and Foxp3 (red). Scale bars, 100 μm **(D)**. Changes in the expression of Foxp3 marker in matched pre- and post-therapy lesions are shown in graphs for *all patients* (*n* = 12, *p* = 0.2334) as well as for the two defined patient sub-groups (*naïve/CT/RT n* = 9, *p* = 0.7334, *immuno_treated n* = 3, *p* = 0.2500) **(E)**. CR and PR Patients are displayed in green, SD in black, and PD in red. Open circles denote on-treatment samples, open triangles post-treatment ones. Statistical comparisons are based on the non-parametric two-tailed Wilcoxon signed-rank test. Only values statistically significant are reported.

To further understand the activation extent of tumor-infiltrating CD8^+^ T cells we examined paired pre and post DC vaccine tumor samples for the presence of Granzyme B (GZMB), a key functional marker of effector cytotoxic CD8^+^ lymphocytes. Thirteen cases were assessable for the GZMB staining. Co-expression of GZMB and CD8 was assessed by sequential IHC, and confirmed that a considerable fraction of CD8^+^ T cells were activated cytotoxic CD8^+^ lymphocytes, rather than potentially activated GZMB^+^CD8^−^ natural killer cells. A representative image of the reconstructed double pseudo-fluorescence image is shown in Figure 3A (Pt#16). Moreover, the tight association of apoptotic or necrotic tumor cells with CD8^+^ TILs together with the polarization of the cytotoxic granules toward melanoma cells (data not shown), strongly support the functional relevance of the defined phenotype. The GZMB:CD8 ratio was used to define the effective fraction of intratumoral cytotoxic T cells. Unexpectedly, no significant change in the amount of GZMB^+^ cells was found between pre- and post-treatment tumor biopsies (mean ± SEM 118.3 ± 47.68 vs. 137.2 ± 51.98 GZMB^+^ cells/mm^2^ in pre-treatment and post-treatment biopsies, respectively; *p* = 0.5830; Figure 3B). In addition, the observed increase in the number of CD8^+^ cells in the *naïve/CT/RT group* was not paralleled by a concurrent increase in GZMB-expressing cells, suggesting that cytotoxic activation of vaccine-induced intratumoral CD8^+^ T cells may have been hampered by the up-regulation of PDL1 on tumor cells. Supporting this hypothesis, we found a significant inverse correlation (Spearman *r* = −0.8667, *p* = 0.0045) between the GZMB:CD8 ratio and the percentage of PDL1^+^ cells (Figure 3C). An additional crucial mechanism involved in impairing the CD8 effector program is the presence in the TME of immune suppressive cells, like Foxp3^+^ regulatory T cells (Tregs). In this respect, we evaluated the density of intratumoral Foxp3^+^ cells in our series. Sequential IHC stainings (Figure 3D) showed that Foxp3 was expressed exclusively in the CD4^+^ compartment, thus excluding any association with CD8 potentially accounting for early effector CD8^+^ T lymphocytes (28) and confirming that in our samples Foxp3^+^ cells were for the large majority Tregs. Due to the shortage of tumor material, four samples could not be assessed (Supplementary Table 1). In the remaining 12 patients we observed a trend (7 out of 12 cases) toward a decrease in the number of Foxp3^+^ cells (mean ± SEM 163.7 ± 101.7 vs. 110.5 ± 44.56 Foxp3^+^ cells/mm^2^ in pre-treatment and post-treatment biopsies, respectively; *p* = 0.7344, Figure 3E). Similarly, no significant increase of this immunosuppressive population was observed in any of the analyzed groups (Figure 3E), indicating that this mechanism is unlikely involved in decreasing cytotoxic activation of CD8^+^ effector cells.

## Discussion

It is widely recognized that DC vaccination as monotherapy has a limited clinical efficacy, particularly in heavily pretreated Patients. However, DC vaccines are very well-tolerated, and in the actual clinical scenario they could have a role in combination immunotherapy with immune checkpoint inhibitors. Nowadays, their interaction is still poorly understood and clinical trials are needed to identify the best sequential or combination regimen. In this respect, immune monitoring within the TME has been fundamental for the discovery of mechanisms of response and resistance to treatment, as well as instrumental for the design of combination regimens to enhance anti-tumor immunity and clinical responses. Accordingly, this study describes the qualitative and quantitative changes of immune cell subpopulations occurring in the TME in a cohort of metastatic melanoma patients treated with autologous tumor lysate loaded mature DCs for whom matched pre and post-therapy material was available from our Institutional repository. Likewise, Gross et al. attempted to score CD3^+^ lymphocytic infiltration upon DC vaccination, but the analysis was conducted on pre-vaccination metastases in 17 patients and post-vaccination metastases in 17 patients, with paired samples available in only seven patients (5). However, albeit limited by the number of samples, a higher lymphocytes score was recorded in post-vaccine tumor samples compared to pre-vaccine ones. Additionally, a higher immune infiltration following DC vaccination was also highlighted in glioblastoma multiforme, particularly including a CD8^+^ T cell population (29). To the best of our knowledge, our study represents the largest retrospective analysis of a unique cohort of matched pre and post samples from metastatic melanoma patients treated with an autologous DC vaccine. Remarkably, our quantitative analysis was conducted on WSI, rather than selected areas, and using resected surgical specimens, which are more representative of the entire TME compared to core needle biopsies or frequently used tissue microarrays. Our data showed that DC vaccination increases the number of intratumoral CD8^+^ T cells, although the differences observed between pre-treatment and post-treatment biopsies were not statistically significant in the whole series. However, when patients were analyzed according to the type of treatment they received before DC vaccination some differences emerged. In particular, patients who previously failed immunological treatments did not show significant changes in the density of intratumoral CD8^+^ T cells, suggesting that the DC vaccine-driven effects on the TME might have been hampered by different mechanisms of immune escape that have led to the failure of previous immunotherapy. Indeed, it has been extensively reported that in the metastatic disease setting, one of the major obstacles to DC vaccine efficacy is represented by the occurrence of multiple mechanisms of tumor-induced immunosuppression (30). Accordingly, it can be assumed that, upon failure to the previous immune-based treatments, patients belonging to the *immuno_treated group* could have developed strong immunosuppression, which negatively affects the ability of DC vaccination to increase intratumoral CD8^+^ T cells, as instead observed in the *naïve/CT/RT group*. Besides not being forms of immunotherapy, we know that chemotherapy (CT) and radiotherapy (RT) might exert immune-modulating effects (31, 32). In light of this, while in all the naïve patients (*n* = 6) we observed a marked CD8^+^ T cell up-regulation compared to baseline treatment (Supplementary Table 1), one CT-treated patient (Pt#7) did not actually display a CD8^+^ T cell increase, thus behaving more similar to the *immune_treated group*. The second striking effect we observed after DC vaccination was the marked up-regulation of PDL1 expression. Similar to what previously described (33) and in accordance to its inducible profile, we found that the intratumoral PDL1 expression was a reflection of the endogenous CD8^+^ T cell abundance, as shown by the positive correlation between the percentages of PDL1^+^ cells and CD8^+^ T cell density. Again, WSI distribution analyses showed a strictly related spatial distribution of PDL1^+^ cells and CD8^+^ TILs. Multiplex IHC for CD45 and PDL1 further confirmed that PDL1 was largely expressed in CD45^−^ tumor cells in close proximity to CD45^+^ immune cells. Our data are also consistent with the hypothesis that cytotoxic activation of CD8^+^ T cells recruited in the TME after DC vaccination can be strongly inhibited by PDL1 concurrently induced in tumor cells, as suggested by the significant negative correlation between PDL1^+^ cells and the GZMB:CD8 ratio. A comprehensive evaluation of the tolerability and clinical efficacy of our DC vaccination protocols has been already provided (4), and an association of the immune contexture with the clinical activity was out of the scope of the current study. Interestingly, in three out of the four truly progressing patients (Pt#13, Pt#14, and Pt#16), the strong rise of CD8^+^ T cells in the tumor microenvironment was matched by an increased expression of the PDL1 inhibitory molecule, thus suggesting an immune escape–associated progression. Although the underlined PDL1 pattern partially explains the limited functionality of intratumoral T cells, we recognize that changes in the immune signature observed did not fully correlate with the clinical outcome of the vaccination. In addition, the lack of correlation between Foxp3^+^ cell densities and cytotoxic activation of CD8^+^ T cells further reinforces the role of PD1/PDL1 axis activation in suppressing DC vaccine-induced cytotoxic immune response. Additional studies, albeit hampered by the limited accessibility to this type of samples, will be needed to confirm our findings and potentially also shed light on other markers/immune populations. Indeed, we do not exclude that other phenomena, such as the variation in the number of intratumoral Foxp3^+^ cells, be involved in the limited clinical efficacy of DC vaccination, and could have been detected if the analysis was conducted on a greater number of cases. DCs by nature are crucial for immunosurveillance and thus more likely for the *de novo* induction of anti-tumor immunity, although DC vaccination could hardly overcome profound tumor-induced immunosuppression. Accordingly, DC vaccination monotherapy is increasingly utilized in the adjuvant setting (34, 35). Conversely, it has been shown that pre-existing spontaneous immune response largely directed against neoantigens are frequently associated with a “hot” (i.e., T cell inflamed) TME, characterized by high levels of CD8^+^ T cells together with immune-mediated adaptive PDL1 up-regulation. Of note, T cell inflamed tumors more likely respond to therapeutic PD-1 blockade (36). Interestingly, a recent phase 3 clinical trial with the combination of the anti-CTLA4 monoclonal antibody ipilimumab and the anti-PD1 nivolumab in metastatic melanoma showed an advantage, both in terms of PFS and OS, of the combination over nivolumab alone in patients with PDL1 negative tumors (37), but at the expenses of much higher toxicity. In this line, synergism might be observed between PD1/PDL1 blockade and treatments up-regulating PDL1 expression in the TME in patients carrying PDL1 negative melanomas. On these grounds, the very favorable toxicity profile, together with its ability to turn “cold” into “hot” tumors, define DC vaccination as a promising candidate for combination with inhibitors of the PD1/PDL1 immune checkpoint.

## Data Availability Statement

All raw data to the main and supplementary data figures are provided in an anonymous format as a FigShare Collection named “2019_BulgarelliEtAl_Patients_Figures”, available at https://doi.org/10.6084/m9.figshare.c.4584041.v3.

## Ethics Statement

The studies involving human participants were reviewed and approved by Comitato Etico della Romagna (CEROM). The patients/participants provided their written informed consent to participate in this study.

## Author Contributions

JB and MT conceived the study, performed the experiments, analyzed and interpreted the data, and wrote the manuscript. AG helped performing some of the experiments. LR provided support with the clinical data and with critical feedback. SM helped performing WSI quantification with *QuPath* software and sequential IHC. FP and AC developed the corner-based 2D image alignment tool, designed the image processing pipeline, and provided support in image analysis. FR and GG provided support with the clinical data. BV and BL provided the Tissue Microarray and technical feedbacks. MP and EP were responsible of the DC vaccine preparation. VA and GF helped collecting and sectioning the FFPE tumor samples. MF arranged the collection of patient samples. MG conceived the study, interpreted the data, and critically reviewed the manuscript. All authors read and approved the final manuscript.

### Conflict of Interest

The authors declare that the research was conducted in the absence of any commercial or financial relationships that could be construed as a potential conflict of interest.
