# Supplementary material for: Dendritic Cell Vaccination in Metastatic Melanoma Turns “Non-T Cell Inflamed” Into “T-Cell Inflamed” Tumors
Source: Front Immunol. 2019 Oct 9;10:2353. doi: 10.3389/fimmu.2019.02353 (PMC6794451; doi:10.3389/fimmu.2019.02353)
Supplement: Supplementary file 1 [file Data_Sheet_1.docx]

**Supplementary Material**

**
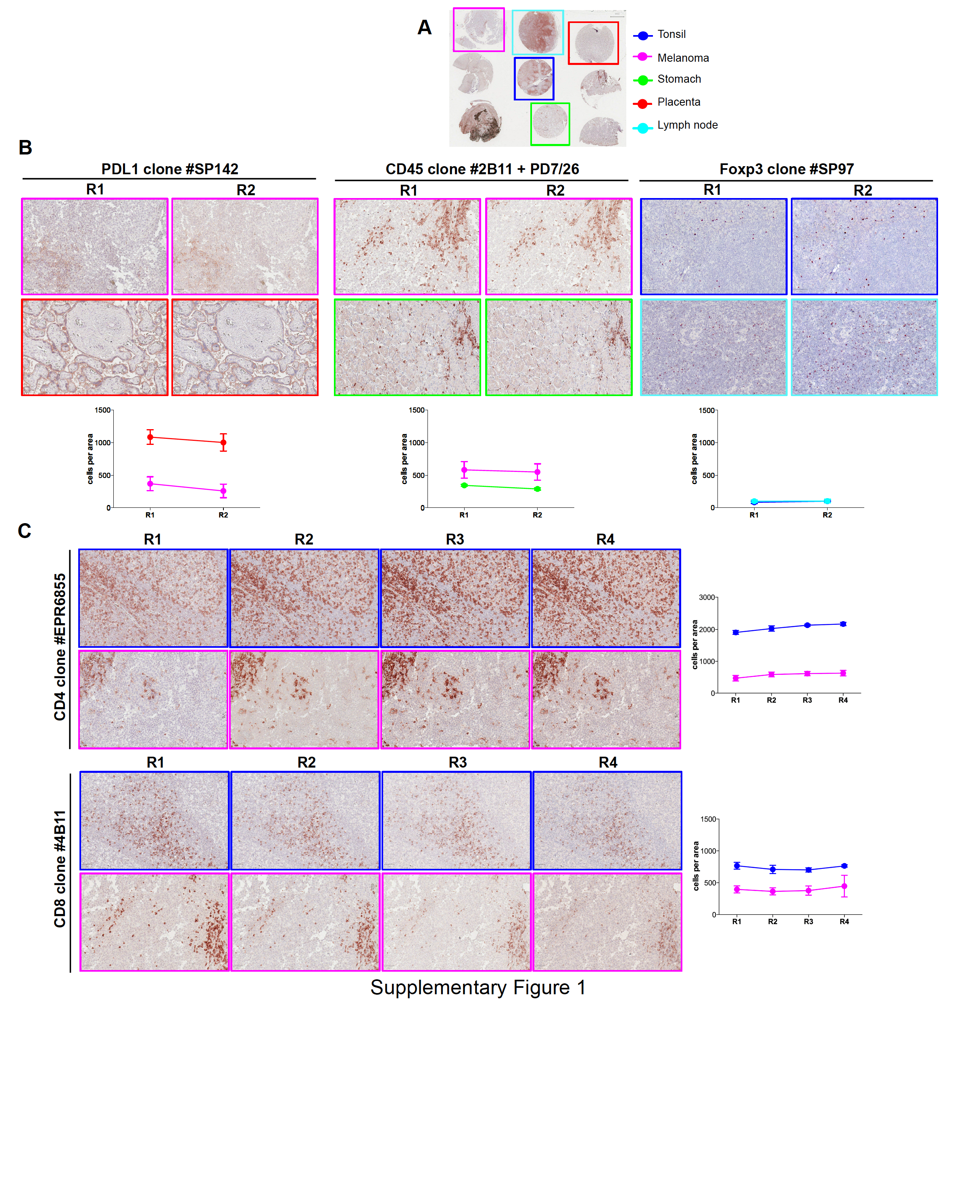
**

**Supplementary Figure 1. Validation of chromogenic sequential IHC.**

FFPE sequential sections from human tissue-assembled TMA including tonsil, melanoma, stomach, placenta and lymph node were used to test different antibodies for sequential IHC **(A)**. PDL1 (clone #SP142), CD45 (clone #2B11+PD7/26) and FOXP3 (clone #SP97) antibodies were tested up to two rounds of staining and representative pictures revealed similar staining intensity. Positive cells for each antibody were enumerated in 3 different areas in at least 2 tissues and the average number of positive cells per round is shown in graphs. The number of detectable PDL1^+^ cells slightly decreases in both tissues (melanoma and placenta) in the second round, while for CD45 and FOXP3 antibodies the average number of detected positive cells is unchanged after the second staining round **(B)**. CD4 (clone #EPR6855) and CD8 (clone #4B11) antibodies were tested up to 4 staining/destaining cycles, representative bright-field images are shown and revealed similar staining intensity. The average number of positive cells for each antibody is shown in graphs and is unchanged among the staining rounds **(C)**. Scale bars, 50µm.


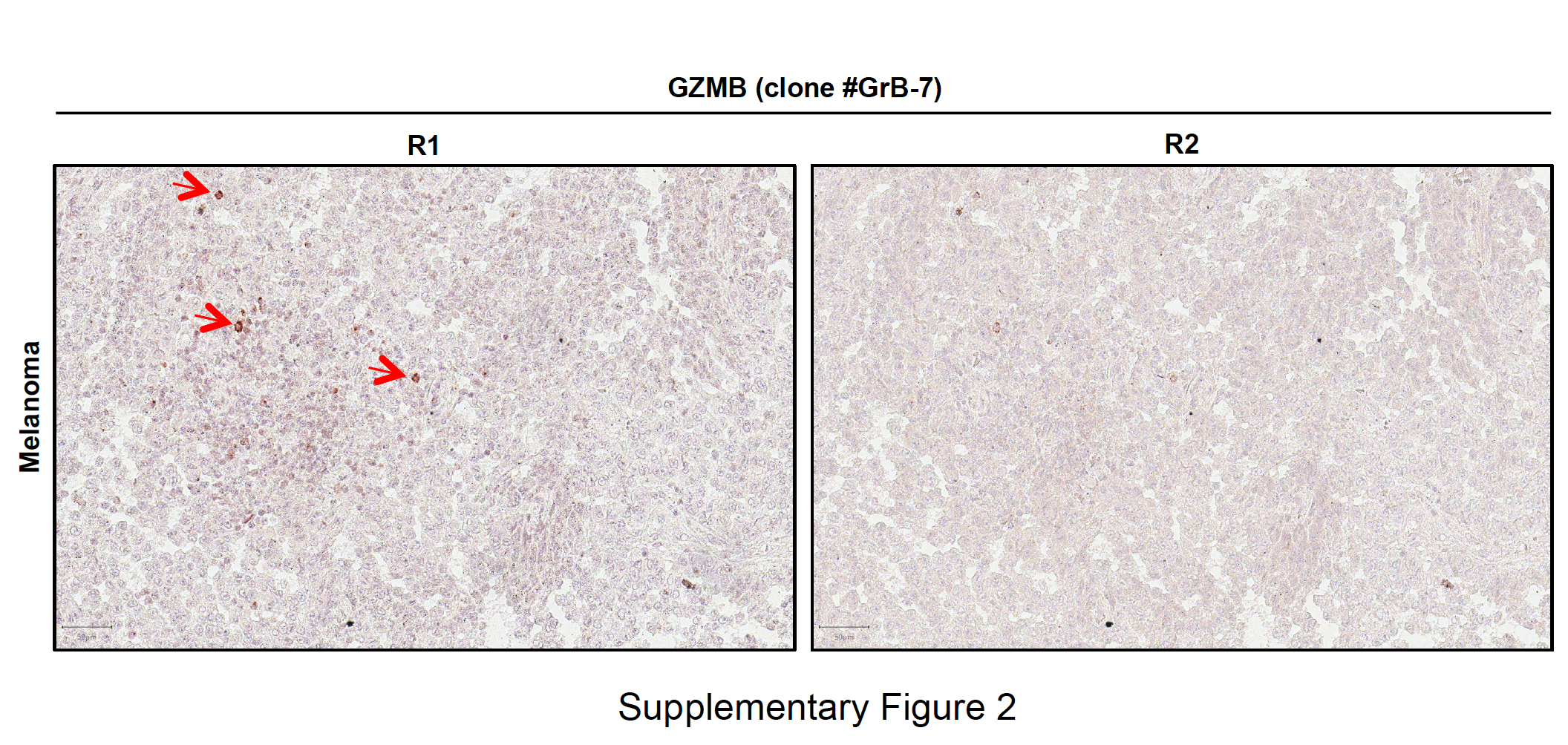


**Supplementary Figure 2. Loss of GZMB staining during sequential IHC.**

Digital scans of bright-field sequential IHC for GZMB (clone #GrB-7) on one FFPE section of human melanoma tissue. Primary antibody was visualized with horseradish peroxidase-conjugated polymer and AEC detection, followed by whole-slide digital scanning. After a destaining step in alcohol gradient and heat-based antibody stripping using citrate (pH 6.0), the sample was restained sequentially with the same antibody. GZMB^+^ cells highlighted by arrows in the first staining round (R1) were almost completely undetectable in the second staining round (R2). For this reason GZMB was always applyed as first staining in sequential IHC. Scale bars, 50µm.

**
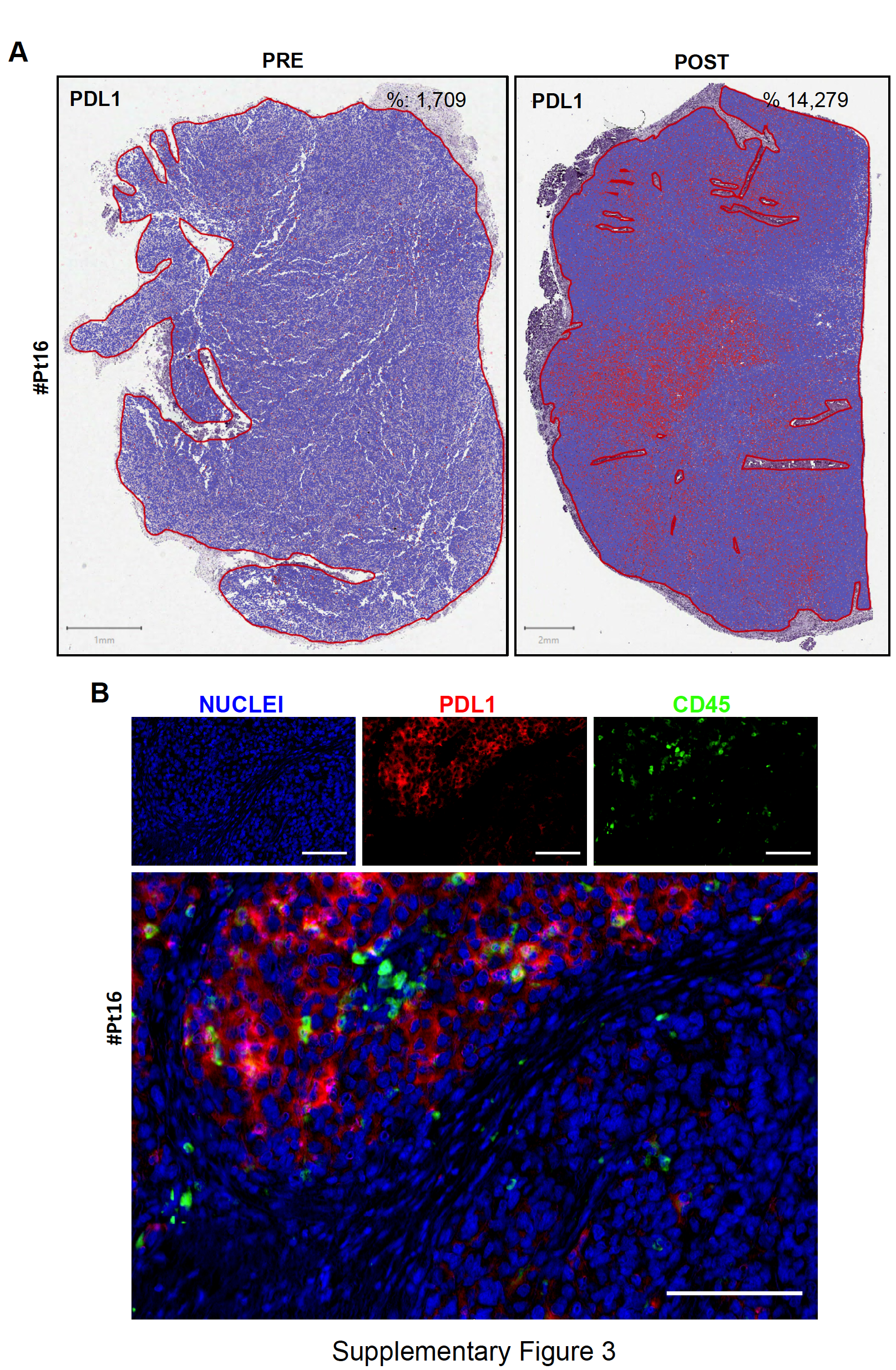
**

**Supplementary Figure 3. Distribution of PDL1^+^ tumor cells and proximity to intratumoral CD45^+^ cells.**

WSIs of PDL1 staining in the matched pre- and post-therapy biopsies of one representative patient (Pt#16) are shown (A). Sequential PDL1 (red) and CD45 (green) IHC staining on the same tissue section highlights the PDL1 expression on tumor cells, and the close proximity between CD45^+^ immune cells and PDL1^+^ tumoral cells. Scale bars, 100µm (B).

**Supplementary Table 1.**

| **Pt#** | **CD4 (cells/mm^2^)** | | **ratio post/pre CD4** | **CD8 (cells/mm^2^)** | | **ratio post/pre CD8** | **Foxp3 (cells/mm^2^)** | | **Foxp3:CD4 ratio** | | **GZMB (cells/mm^2^)** | | **GZMB:CD8 ratio** | | **PDL1% in total cells** | |
| --- | --- | --- | --- | --- | --- | --- | --- | --- | --- | --- | --- | --- | --- | --- | --- | --- |
|  | **pre** | **post** |  | **pre** | **post** |  | **pre** | **post** | **pre** | **post** | **pre** | **post** | **pre** | **post** | **pre** | **post** |
| 1# | 2037,501 | 614,434 | **0,302** | 1849,423 | 2035,979 | **1,101** | 968,049 | 444,686 | 0,475 | 0,724 | 416,515 | 627,827 | 0,225 | 0,308 | 7,836 | 3,022 |
| 2# | na | 347,092 | **na** | 369,043 | 337,030 | **0,913** | 8,330 | na | na | na | 28,954 | 86,290 | 0,078 | 0,256 | 0,726 | 0,402 |
| 3# | 547,120 | 308,361 | **0,564** | 1022,991 | 842,387 | **0,823** | 42,150 | 27,480 | 0,077 | 0,089 | 607,983 | 517,954 | 0,594 | 0,615 | 16,750 | 6,185 |
| 4# | 379,841 | 355,511 | **0,936** | 1068,927 | 185,006 | **0,173** | na | 51,279 | na | 0,144 | 77,878 | 0,606 | 0,073 | 0,003 | 4,744 | 4,793 |
| 5# | 90,910 | 1384,630 | **15,231** | 1244,522 | 1665,079 | **1,338** | 54,804 | 62,747 | 0,603 | 0,045 | 63,752 | 81,546 | 0,051 | 0,049 | 11,130 | 24,700 |
| 6# | 257,281 | na | **na** | 558,959 | 291,099 | **0,521** | na | na | na | na | 0,677 | 1,345 | 0,001 | 0,005 | 0.9748 | 9,203 |
| 7# | na | na | **na** | 500,558 | 280,169 | **0,560** | 49,568 | 33,442 | na | na | 27,848 | 188,379 | 0,056 | 0,672 | 0,826 | 7,519 |
| 8# | na | 100,607 | **na** | 172,405 | 518,915 | **3,010** | 52,559 | 106,760 | na | 1,061 | 97,007 | 5,123 | 0,563 | 0,010 | 0,000 | 33,639 |
| 9# | na | na | **na** | 79,074 | 319,415 | **4,039** | 13,384 | 13,287 | na | na | 23,456 | 49,703 | 0,297 | 0,156 | 0,602 | 6,530 |
| 10# | 261,990 | 98,952 | **0,378** | 107,086 | 11,758 | **0,110** | 64,842 | 10,657 | 0,247 | 0,108 | 17,427 | na | 0,163 | na | 8,771 | na |
| 11# | 514,011 | 293,634 | **0,571** | 282,528 | 297,033 | **1,051** | 177,486 | 108,357 | 0,345 | 0,369 | 14,052 | 10,077 | 0,050 | 0,034 | 1,255 | 13,999 |
| 12# | 1742,237 | na | **na** | 1300,553 | 1762,566 | **1,355** | 231,715 | 43,759 | 0,133 | na | na | na | na | na | na | na |
| 13# | 189,806 | 39,182 | **0,206** | 215,358 | 486,609 | **2,260** | 36,914 | 15,240 | 0,194 | 0,389 | 35,475 | 65,396 | 0,165 | 0,134 | 1,677 | 8,201 |
| 14# | 241,223 | na | **na** | 186,040 | 932,611 | **5,013** | 64,934 | 152,270 | 0,269 | na | 202,525 | 86,870 | 1,089 | 0,093 | 2,465 | 11,765 |
| 15# | 98,696 | 8,687 | **0,088** | 339,784 | 366,900 | **1,080** | na | 76,751 | na | 8,835 | 45,690 | 33,412 | 0,134 | 0,091 | 2,379 | 0,893 |
| 16# | 241,024 | 832,408 | **3,454** | 269,037 | 1371,354 | **5,097** | 55,293 | 57,908 | 0,229 | 0,070 | 13,928 | 166,965 | 0,052 | 0,122 | 1,709 | 14,279 |

Summary of IHC results. Abbreviations: Pt, patient; na, not assessable.
